# Case Report of Wound Treatment with Hyiodine Gel in an Occasional KID Syndrome Patient

**DOI:** 10.3390/jcm15010011

**Published:** 2025-12-19

**Authors:** Marianna Hajská, Silvia Bittner Fialová, Martin Dubovský, Arpád Panyko

**Affiliations:** 1 Comenius University Bratislava, Faculty of Medicine and University Hospital, 4th Department of Surgery, Bratislava, Ružinovská 6, 82606 Bratislava, Slovakia; marianna.hajska@fmed.uniba.sk (M.H.); martin.dubovsky@fmed.uniba.sk (M.D.); arpad.panyko@fmed.uniba.sk (A.P.); 2 Comenius University Bratislava, Faculty of Pharmacy, Department of Pharmacognosy and Botany, Odbojárov 10, 83232 Bratislava, Slovakia

**Keywords:** keratitis–ichthyosis–deafness (KID) syndrome, chronic wounds, surgical debridement, hyperkeratosis, multidisciplinary management

## Abstract

**Background/Objectives**: Keratitis–ichthyosis–deafness (KID) syndrome is an exceptionally rare congenital multisystem disorder, with an estimated prevalence below 1:1,000,000 and fewer than 100 reported cases worldwide. It is characterized by hyperkeratosis, alopecia, nail dystrophy, hearing loss, and ocular involvement. While dermatological management is well described, surgical experience with wound treatment in KID syndrome remains extremely limited. The objective of this report is to describe the surgical management and outcomes of a patient with chronic lower-limb wounds associated with KID syndrome. **Methods**: A 35-year-old male with KID syndrome was referred to the surgical outpatient clinic for chronic traumatic wounds of both lower limbs resistant to conservative dermatological therapy. Initial outpatient treatment included serial sharp surgical debridement under local anesthesia, combined with topical keratolytics and silver sulfadiazine with hyaluronic acid. Due to minimal improvement after three months, an inpatient surgical approach was initiated, involving complete excision of hyperkeratotic tissue, creation of a wide ulcer bed, and adjunct systemic and topical therapies. **Results**: The inpatient management enabled thorough removal of pathological tissue and better control of local infection and inflammation. Combined systemic antibiotic and antipsoriatic therapy, together with topical sodium hyaluronate and iodine, was associated with gradual wound healing and improved skin condition. The patient tolerated the procedures well, without major complications. **Conclusions**: Surgical debridement and excision, when combined with targeted dermatological and antimicrobial therapy, can be an effective and safe strategy for managing chronic wounds in KID syndrome. This case highlights the importance of multidisciplinary collaboration and individualized surgical planning in this extremely rare disorder.

## 1. Introduction

Keratitis–ichthyosis–deafness (KID) syndrome is a sporadic genetic disorder with an estimated prevalence below 1:1,000,000, and approximately one hundred patients have been described in the literature [1]. The disease results from heterozygous mutations in the GJB2 gene encoding connexin-26 (Cx26), a structural component of gap junction channels responsible for ionic and metabolic intercellular communication [2]. The GJB2 gene is expressed in a variety of tissues, including several ectodermal epithelia affected in KID syndrome: the corneal epithelium, epidermis of skin, cochlea, and hair follicles [3]. Pathogenic mutations lead to hemichannel dysfunction and impaired keratinocyte differentiation, clinically manifesting as hyperkeratosis, impaired skin barrier, inflammatory complications, and frequent infections. The classical triad includes congenital sensorineural hearing loss, ichthyosis, and progressive keratitis, with cutaneous involvement being the dominant phenotype in most patients [4]. Typical findings include diffuse erythematous thickening of the skin with pronounced hyperkeratosis—particularly on palms, soles, and lower legs—dry scaly skin, hyperkeratotic plaque-like lesions, and papillomatous deposits. Nail dystrophy, paronychia, alopecia, and loss of eyebrows or eyelashes are common. Ophthalmological involvement presents photophobia, recurrent keratitis, and progressive corneal vascularization, potentially resulting in severe visual impairment requiring keratoplasty [5]. Ocular lesions arise later in KID syndrome than the other abnormalities, and symptoms may not be present until adolescence [6]. Genodermatoses, such as keratitis–ichthyosis–deafness syndrome, can occasionally be associated with hidradenitis suppurativa (HS) and HS-related cutaneous squamous cell carcinoma [7]. Recent literature further expands the phenotypic spectrum of KID syndrome beyond the classical triad. Several case reports also document neurological, skeletal, and systemic abnormalities, underscoring the multisystem consequences of GJB2-related channel dysfunction. For example, ventriculomegaly, seizures, developmental delay, congenital muscular torticollis, and hip dysplasia have been reported in a Jordanian child with KID syndrome, suggesting broader ectodermal and neurodevelopmental involvement [8]. Overall prognosis is favorable except for the risk of development of squamous cell carcinoma (SCC) of the mucosa of the eye and oral cavity [9].

Disrupted skin barrier function, hyperkeratosis, and frequent fissures predispose these patients to chronic bacterial and fungal infections. Reported pathogens include *Staphylococcus aureus*, *Streptococcus* spp., *Proteus mirabilis*, *Pseudomonas aeruginosa*, *Escherichia coli*, *Klebsiella pneumoniae*, and *Candida albicans* [1]. Infections may remain localized, but severe systemic complications, including sepsis, have been reported, sometimes refractory to intensive therapy [2]. The diagnosis and treatment of skin superinfections in KID syndrome patients is challenging [10]. Beyond increased susceptibility to cutaneous infections, systemic infectious complications such as endocarditis and parainfectious myelitis have been documented, illustrating the extent to which impaired skin barrier function facilitates the dissemination of pathogens such as *Staphylococcus aureus* [11]. Healing of chronic ulcerations is hindered by thick keratotic layers, which act as a physical barrier to spontaneous epithelialization and limit penetration of topical agents. Treatment often employs emollients, keratolytics, antiseptics, and topical antimicrobials, yet outcomes are frequently only partial [1]. Existing literature suggests treating the secondary infection with hydrosurgery, which supports antibacterial and antifungal therapy, silver-containing dressings, and a cream containing *Gentiana purpurea* [12]. Another alternative may be natural products [13]. Kanuka honey, for instance, has been reported as potentially beneficial for the treatment of actinic keratosis, with no recurrence observed following a three-month therapy period [14]. *Aloe vera* [15], hyaluronic acid [16,17], acetic acid [18], ginsenosides [19], and *Ginkgo biloba* [20] are currently being investigated for their ability to enhance skin structure or hydration in hyperkeratotic conditions.

Except for topical treatment, most published reports focus on general pediatric management. Despite increasing recognition of the multisystem nature of KID syndrome, published surgical experience remains extremely limited, which complicates clinical decision-making in cases requiring debridement or excision. Montanari et al. recently reported a case of a 7-year-old boy with dyskeratotic neoformations requiring surgical excision. Postoperative complications included dehiscence, infection, and the need for NPWT (Negative Pressure Wound Therapy) and topical honey-based products. Kapila et al. described a pediatric patient with extensive purulent papillomatous hyperkeratoses requiring hospitalization and repeated surgical debridement; massive intraoperative blood loss after hydrosurgical debridement required transfusions and ICU (Intensive Care Unit) admission. Both reports emphasize that repeated debridement, removal of necrotic and keratotic layers, and adequate wound coverage may be necessary to achieve local wound stabilization. These findings suggest that although surgical treatment is feasible, it is highly demanding and requires multidisciplinary management, aggressive antimicrobial prophylaxis, and advanced wound care. Another rare case, also reporting a beneficial effect of surgical debridement, was published recently and described a 17-year-old female patient with a known diagnosis of KID who presented with refractory wounds on the capillitium. Her cranial magnetic resonance imaging (cMRI) scan revealed chronic osteomyelitis of the skull bone corresponding to an active abscess within the frontal scalp. Surgical debridement and abscess evacuation were performed multiple times, and the authors observed that surgical debridement significantly reduced pain and improved cervical mobility, which had been restricted by scar tissue [21].

## 2. Detailed Case Description

We report the case of a 35-year-old male patient with a genetically confirmed diagnosis of keratitis–ichthyosis–deafness (KID) syndrome caused by a heterozygous GJB2 mutation (p.D50N). According to available national data, he represents the only documented patient with this specific mutation in Slovakia. Since early childhood, he manifested severe and persistent cutaneous features characteristic of KID syndrome, including diffuse hyperkeratosis, extensive keratotic plaques, deep fissuring, recurrent inflammatory changes, and frequent secondary infections affecting wide areas of the integument. He remained under long-term dermatological follow-up, receiving continuous topical and systemic therapy aimed at improving the skin barrier and controlling infectious complications. In December 2022, the patient was referred to our surgical outpatient clinic for chronic, non-healing traumatic wounds of both lower limbs, which developed after a bicycle fall and did not respond to standard dermatological management. Before his injury, the patient was professionally active, fully independent, and physically fit; however, progressive wound deterioration, severe pain, and reduced mobility profoundly impaired his ability to work and participate in daily activities, leading to significant social and functional decline. During the initial phase of treatment, when the patient was managed in the outpatient setting, he attended regular follow-up visits. Each visit included a structured clinical examination, wound evaluation, and microbiological sampling by wound swabs. We also reviewed dermatology reports on every visit and maintained close communication with the patient’s dermatologist to coordinate care. To control hyperkeratosis and reduce the infectious load, repeated sharp surgical debridement of smaller wound areas was attempted. However, the procedure was significantly limited by the patient’s pronounced pain sensitivity, despite local anesthesia. Topical therapy consisted of sterile gauze dressings impregnated with 1% acetic acid and a cream containing silver sulfadiazine, supplemented by keratolytic agents. Despite adherence to therapy and multidisciplinary coordination, the wounds showed minimal improvement, and the patient developed increasing functional limitations. Given the severity of hyperkeratosis, the failure of outpatient debridement due to pain intolerance, and the progressive functional decline, radical tangential excision was selected as the only feasible surgical strategy. This decision was made with awareness of the increased bleeding risk described in previous KID syndrome cases. The team therefore planned staged procedures under spinal anesthesia with immediate availability of blood products and postoperative monitoring to minimize complications.

Upon hospital admission, the patient was afebrile, hemodynamically stable, and showed no signs of systemic infection. Examination of the lower limbs revealed extensive hyperkeratosis with large ulcerations on both shins. The wound surfaces were overlaid with thick, avascular keratotic masses and purulent discharge, while the surrounding tissue exhibited chronic inflammatory changes. Pain was elicited primarily during palpation and movement. Laboratory tests showed normal leukocyte counts and a moderately elevated C-reactive protein level (22 mg/L). Repeated blood cultures remained negative. Given the chronicity and severity of the lesions, a staged surgical approach was undertaken. Three wide necrosectomies were performed under spinal anesthesia at seven-day intervals. Sharp tangential excision using a Humby knife was selected as the optimal technique to allow controlled and sequential removal of devitalized keratinous and necrotic tissue while preserving viable structures. The procedures were performed first on the right leg, then the left, and finally targeting residual hyperkeratotic areas bilaterally. This radical removal of pathological tissue eliminated the mechanical barrier preventing epithelial migration, reduced the microbial load, and enabled effective penetration of topical agents. Between interventions, daily wound care was conducted by physicians following principles analogous to deep burn management, reflecting the macroscopic appearance of the defects. After careful irrigation with antiseptic solution, paraffin gauze dressings were applied as a non-adherent contact layer, followed by sterile dressings impregnated with 1% acetic acid and silver sulfadiazine with sodium hyaluronate. Early postoperative evaluations revealed unexpectedly strong regenerative capacity of the wound bed. To optimize moist wound healing, minimize cumulative exposure to silver, and support granulation, local therapy was subsequently transitioned to a gel containing 1.5% hyaluronic acid and a 0.25% iodine complex (Hyiodine^®^). Serial microbiological cultures repeatedly confirmed polymicrobial colonization (Table 1), including *Proteus mirabilis*, *Pseudomonas aeruginosa*, methicillin-susceptible *Staphylococcus aureus*, *Corynebacterium striatum*, *Enterococcus avium*, and *Candida albicans*. Histopathological examination demonstrated dyskeratotic changes consistent with KID syndrome and superficial fungal involvement, justifying the introduction of targeted antibacterial and antifungal systemic therapy coordinated with a clinical pharmacologist.

The surgical procedures were technically demanding and associated with substantial intraoperative bleeding during the first two tangential excisions—hemoglobin levels decreased from 128 g/L to 86 g/L—necessitating transfusions, although the patient remained hemodynamically stable and never required intensive care admission. Postoperatively, the wounds showed progressive granulation, a marked reduction in secretion, resolution of malodor, and declining inflammatory signs. Shortly before discharge, the patient developed a transient fever; a comprehensive diagnostic evaluation ruled out infection, and impaired thermoregulation related to the underlying genodermatosis was considered the most likely cause. The patient tolerated the multimodal treatment well, without local or systemic adverse reactions. Laboratory parameters remained stable throughout hospitalization. After extensive interdisciplinary evaluation, he was discharged with detailed wound-care instructions and scheduled outpatient follow-up. Following appropriate education, he became nearly independent in performing dressing changes at home.

**Figure 1:**
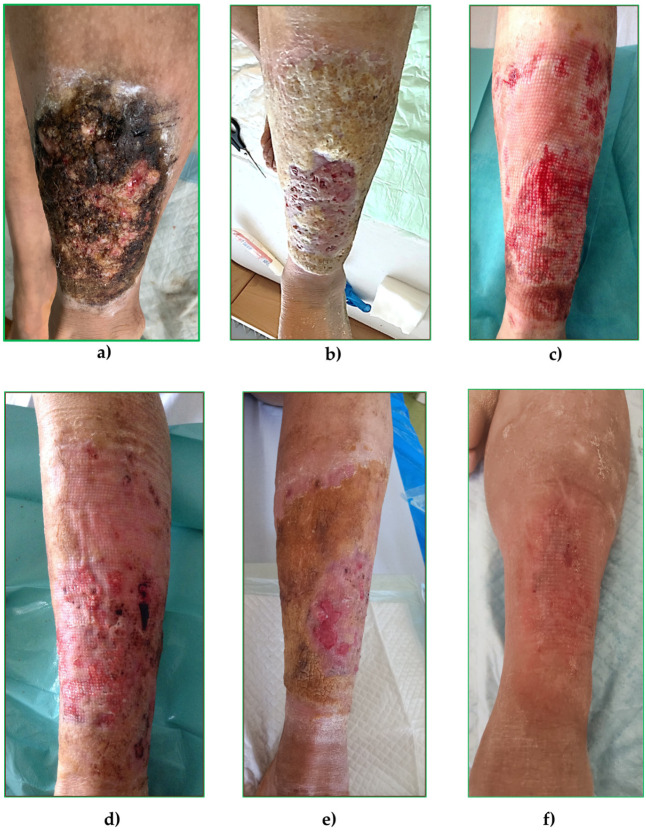
Changes in wound surface appearance during wound care in KID syndrome patient: (**a**) after injury, (**b**) before surgical debridement, (**c**) day 2 after surgery, (**d**) day 14 after surgery, (**e**) one month after surgery, and (**f**) 10 months after surgery.

Within the first month, wound status improved substantially, pain levels decreased, and mobility increased. Three months after the initial hospitalization, a brief elective readmission was required for minor debridement of a superficial recurrent lesion on the left leg, which healed uneventfully under continued topical therapy. After more than two years of follow-up, the patient achieved stable wound closure, full functional recovery, and successful reintegration into work and recreational activities, choosing running instead of cycling. Photographic documentation is shown in Figure 1. This case demonstrates that a combination of radical surgical debridement, targeted pharmacological management, and sustained interdisciplinary collaboration can lead to successful long-term outcomes in patients with extremely rare and complex genodermatoses such as KID syndrome. While the observed postoperative course was favorable, interpretation of the treatment effect is inherently limited by the single-patient design and absence of comparative data, particularly regarding the relative contribution of surgery versus systemic or topical therapy.

### Pharmacological Treatment

Management required close collaboration with a clinical pharmacologist. Targeted antimicrobial therapy was initiated: ciprofloxacin initially, then switched to doxycycline combined with itraconazole after confirmation of fungal infection. Long-term systemic acitretin, antacids, analgesics as needed, and specialized topical therapy, primarily hyaluronic acid, were continued. Local topical treatment formed a key role in complex care. All the topical antimicrobials used in this case are listed below.

Pre-surgery and immediately after tangential excision:

Contact layer—Paraffine gauze dressing Lomatulle^®^ H (Lohmann & Rauscher GmbH & Co., KG, Neuwied, Germany).

Antimicrobial layer—sterile gauze impregnated with acetic acid 1% solution (magistraliter) and Ialugen^®^ Plus (IBSA Farmaceutici Italia Srl, Lodi, Italy).

Subsequent local care:

Gel containing 1.5% hyaluronic acid and 0.25% iodine complex—Hyiodine^®^ (Contipro, Dolní Dobrouč, Czech Republic).

## 3. Discussion

KID syndrome is a rare genetic disorder with severe impairment of the skin barrier, predisposition to hyperkeratosis, ulceration, and polymicrobial infection. Wounds in these patients have a unique pathophysiology—keratotic layers create a mechanical barrier to healing, promote biofilm formation, and limit penetration of topical agents. Standard conservative therapy is frequently ineffective, resulting in chronic non-healing defects. Our team recently had the opportunity to provide complex wound care to the patient with this rare disease, which we consider extremely challenging because of no previous experience and very limited literature evidence of soft tissue surgery in KID syndrome patients. In our case, wound treatment of chronic defects of former traumatic origin located on both lower limbs, refractory to standard conservative treatment in the markedly hyperkeratotic tissue, was performed, including both surgical and conservative therapy. During the initial period provided in the outpatient department, a combination of acetic acid and silver sulfadiazine cream was applied locally. Unfortunately, topical therapy did not bring sufficient healing outcome, and more therapeutic options were discussed, both within our clinician team and with the patient and his mother as well. Considering the recommendations of Kapila and Höner [21,22], we decided to perform surgical debridement. To provide complex care, optimal pain treatment, and be prepared for complications like severe bleeding, the patient was admitted to the hospital. Surgical debridement was performed using tangential excision of necrotic tissue. Thereafter, the question of ideal local treatment had to be solved. We discussed the strategy of change of the topical antimicrobials. The patient was treated with silver sulfadiazine cream for several weeks, so it was essential to find a non-silver-containing alternative. The absolute need was to select a product with both healing potential and good antimicrobial properties, and we decided to use the wound gel combining hyaluronic acid and iodine. Hyaluronic acid (HA), a naturally occurring glycosaminoglycan found in the extracellular matrix of various tissues [23], plays a role in cell differentiation, embryological development, inflammation, wound healing, and other biological processes [24,25]. Furthermore, HA’s contribution to each stage of normal wound healing has been examined, emphasizing its importance in the wound-healing process [26]. On the other hand, iodine is known for its broad-spectrum antimicrobial activity, effective against a wide range of pathogens, including antibiotic-resistant strains [27,28]. The innovative combination of HA and iodine, particularly in gel form, offers a promising approach, recently described by Forghani et al. [29].

Wound improvement became clinically apparent immediately after necrectomy, suggesting that removal of hyperkeratotic infected barriers was the primary driver of healing. Subsequent application of the hyaluronic acid–iodine gel appeared to support further progress, although the relative contribution of individual modalities cannot be determined in a single case. This case demonstrates that surgical debridement may be a key therapeutic step. Removal of avascular and infected keratotic layers enabled restoration of granulation and epithelialization, reduced infectious burden, and ensured adequate efficacy of topical and systemic therapy. Subsequent postoperative wound care contributes essentially to the long-term success of therapy. Closed dressing method with moist wound care is, according to our experience, necessary. Nevertheless, management is challenging, especially since surgical treatment should be performed exclusively in a hospital, because of intraoperative bleeding and transfusion needs. Complex wound care in a patient with KID syndrome requires a combination of surgical intervention, targeted antimicrobial treatment, retinoids, and long-term dermatological follow-up. Our experience aligns with previously reported cases [1,21,22] showing that surgical intervention, although technically demanding and associated with considerable bleeding risk, may be indispensable in reversing chronicity in KID-related ulcerations.

## 4. Conclusions

Our experience confirms that clinical improvement can be achieved even in extremely rare genodermatoses if a multidisciplinary approach is applied, emphasizing radical debridement, infection control, and individualized wound care. It is essential to anticipate long-term treatment, poor efficacy of local anesthesia, significant intraoperative bleeding during sharp debridement, and the need for transfusion, as well as altered thermoregulation. In the presented patient, local wound care based on the modern moist-wound-healing principles and products containing hyaluronic acid (e.g., Hyiodine^®^) showed a beneficial effect. This case also highlights the absence of standardized clinical guidelines for surgical wound management in KID syndrome, which is unsurprising given the rarity of the disease. Given the inherent limitations of single-case evidence, the findings should be interpreted cautiously; nevertheless, they highlight practical considerations that may assist clinicians facing similarly complex wound presentations.

## Figures and Tables

**Table 1:** Microbiological findings—wound swabs.

Pathogen	Characteristics
*Proteus mirabilis*	massive growth; susceptible to meropenem, cefotaxime, cefepime, ampicillin-sulbactam
*Pseudomonas aeruginosa*	sparse to moderate growth; susceptible to meropenem, ceftazidime, colistin, and aminoglycosides
*Staphylococcus aureus* (MSSA)	moderate growth; susceptible to oxacillin, cefazolin, vancomycin, linezolid, and tigecycline
*Corynebacterium striatum*	moderate to massive growth
*Enterococcus avium*	moderate to massive growth
*Candida albicans*	susceptible to a broad spectrum of antifungals

## Data Availability

The data presented in this study are available upon request from the corresponding author due to privacy concerns.

## Abbreviations

The following abbreviations are used in this manuscript:

KID	Keratitis–Ichthyosis–Deafness
ICU	Intensive Care Unit
NPWT	Negative Pressure Wound Therapy
MSSA	Methicillin-Susceptible *Staphylococcus aureus*
CRP	C-Reactive Protein
